# Low septal to lateral wall ^18^F-FDG ratio is highly associated with mechanical dyssynchrony in non-ischemic CRT candidates

**DOI:** 10.1186/s13550-019-0575-9

**Published:** 2019-12-09

**Authors:** Ganna Degtiarova, Piet Claus, Jürgen Duchenne, Marta Cvijic, Georg Schramm, Johan Nuyts, Jens-Uwe Voigt, Olivier Gheysens

**Affiliations:** 1 Department of Imaging and Pathology, KU Leuven, Leuven, Belgium; 2 Nuclear Medicine and Molecular Imaging, University Hospitals Leuven, Leuven, Belgium; 3 Department of Cardiovascular Sciences, KU Leuven, Leuven, Belgium; 4 Department of Cardiovascular Diseases, University Hospitals Leuven, Leuven, Belgium

**Keywords:** Mechanical dyssynchrony, Perfusion, Metabolism, Cardiac resynchronization therapy, Positron emission tomography

## Abstract

**Background:**

In order to better understand the concept of mechanical dyssynchrony, a promising hallmark of cardiac resynchronization therapy (CRT) response, we investigated its effect on regional myocardial metabolism and myocardial blood flow (MBF) in non-ischemic CRT candidates.

**Results:**

Thirty consecutive non-ischemic CRT eligible patients underwent static ^18^F-FDG and resting dynamic ^13^N-NH_3_ PET/CT. ^18^F-FDG uptake and MBF for septal and lateral wall were analysed and septal-to-lateral wall ratios (SLR) were calculated. Based on the presence of mechanical dyssynchrony (septal flash and/or apical rocking) on echocardiography, patients were divided into 2 groups, with (*n* = 23) and without (*n* = 7) mechanical dyssynchrony.

Patients with mechanical dyssynchrony had significantly lower ^18^F-FDG SUVmean in the septum compared with the lateral wall (5.58 ± 2.65 vs 11.19 ± 4.10, *p* < 0.0001), while patients without mechanical dyssynchrony had a more homogeneous ^18^F-FDG distribution (7.33 ± 2.88 vs 8.31 ± 2.50, respectively, *p* = 0.30). Similarly, MBF was significantly different between the septal and lateral wall in the dyssynchrony group (0.57 ± 0.11 ml/g/min vs 0.92 ± 0.23 ml/g/min, respectively, *p* < 0.0001), whereas no difference was observed in the non-dyssynchrony group (0.61 ± 0.23 ml/g/min vs 0.77 ± 0.21 ml/g/min, respectively, *p* = 0.16). ^18^F-FDG SLR, but not MBF SLR, was associated with the presence of mechanical dyssynchrony and showed a significant inverse correlation with volumetric reverse remodeling after CRT (*r* = − 0.62, *p* = 0.001).

**Conclusions:**

Non-ischemic heart failure patients with mechanical dyssynchrony demonstrate heterogeneous regional metabolism and MBF compared with patients without dyssynchrony. However, only ^18^F-FDG SLR appeared to be highly associated with the presence of mechanical dyssynchrony.

**Trial registration:**

Clinicaltrials, NCT02537782. Registered 2 September 2015.

## Introduction

During the last decade, ventricular conduction disturbances have been shown to be associated with adverse cardiac remodeling and to contribute to the development of heart failure (HF) and an increased risk of all-cause mortality [1]. Even though cardiac resynchronization therapy (CRT) is a promising therapy for patients with ventricular conduction abnormalities, 30–40% of patients do not respond to this treatment and therefore optimization of selection criteria for CRT candidates is still an active area of investigation [2]. Along with electrical dyssynchrony induced by inhomogeneous electrical activation of the left ventricle (LV), about 60–70% of patients also develop mechanical dyssynchrony, characterized by discoordinate myocardial deformation and inefficient contraction [3]. Septal flash (SF) and apical rocking (AR), surrogate markers of mechanical dyssynchrony, have been shown to be associated with favorable CRT response [3]. Deeper insights into pathophysiological processes related to mechanical dyssynchrony are needed, including changes in perfusion and metabolism, in order to better understand the adverse cardiac remodeling and to further improve patient selection for CRT.

Different non**-**invasive imaging techniques are currently available to evaluate regional changes in perfusion and metabolism and their interplay. ^18^F-fluorodeoxyglucose (^18^F-FDG) positron emission tomography (PET) studies in patients with ventricular conduction abnormalities have shown regional changes in glucose metabolism with a relatively reduced glucose uptake in the septum compared with an increased uptake in the lateral wall [4, 5].

In contrast to the well-established changes in glucose uptake, data on myocardial perfusion in patients with ventricular dyssynchrony remain controversial. Experimental data have consistently shown a relative hypoperfusion in the septal wall compared with the lateral wall, while clinical studies predominantly reported a rather homogeneous perfusion across the LV, with only few studies showing septal hypoperfusion [4, 6, 7].

Despite several studies investigating myocardial metabolism and perfusion in patients with ventricular conduction abnormalities, data on the influence of mechanical dyssynchrony on these physiologic parameters are lacking.

The aim of our study was to investigate the effect of mechanical dyssynchrony on regional ^18^F-FDG uptake and absolute myocardial blood flow (MBF) (derived from ^13^N-NH_3_ kinetic models) in patients with non-ischemic cardiomyopathy, eligible for CRT.

## Methods

### Study population

Patients with non-ischemic HF referred for CRT implantation were recruited at the University Hospital Leuven and were part of the Leuven cohort of the WORK-CRT study (Clinical trials NCT02537782). Inclusion for CRT was based on the current ESC guidelines [8]. Coronary artery disease was excluded by late gadolinium enhancement cardiac magnetic resonance (CMR) and/or coronary angiography obtained no more than 3 months before CRT implantation and/or thorough evaluation of patient history and complaints by an experienced treating cardiologist. Exclusion criteria comprised the presence of a right bundle branch block, permanent atrial fibrillation/flutter or tachycardia (> 100 bpm), difficulties to obtain LV volumes by echocardiography, history and findings suggestive of ischemic myocardial disease, valve surgery within 90 days prior to enrolment, history of or listing for heart transplantation, implanted LV assist device, severe aortic stenosis, complex and uncorrected congenital heart disease, pregnant and breastfeeding women, and enrollment in one or more concurrent studies that would confound the results of this study. The study was approved by the institutional ethics committee and all patients gave written informed consent prior to inclusion and any study procedure.

### Echocardiography

All patients underwent a standard two-dimensional echocardiography within 1 week before CRT implantation and approximately 12 months after CRT implantation using commercially available systems (Vivid E9 and E95, GE Vingmed Ultrasound, Horten, Norway). Acquired data were stored digitally and analysed off-line using an EchoPAC workstation (version 202, GE Vingmed Ultrasound). LV end-diastolic volume (EDV), end-systolic volume (ESV), and LV ejection fraction (LVEF) were measured using the modified biplane Simpson’s method. Volumetric reverse remodeling after CRT was assessed as the relative change in LV ESV between baseline and post-CRT (Δ LV ESV, %). Mechanical dyssynchrony was visually assessed by two independent readers (JD and MC) on pre-CRT echocardiography images. A third reader (JUV) blinded to previous readings was asked in case of disagreement. Patients were divided into 2 groups—one group with mechanical dyssynchrony defined by the presence of either AR or SF, or both, and another group without mechanical dyssynchrony (neither AR nor SF).

Additionally, pre-CRT echocardiography was used to calculate segmental myocardial work, using an 18 segment model and a method, previously described by our group [9]. In short, a dedicated, MATLAB-based (version 2017b, The MathWorks, Inc., Natick, MA, USA) research software (TVA version 22.00, JU Voigt, Leuven) was used to determine LV pressure estimates according to the method described by Russell [10]. LV segmental mid-wall curvature was dynamically estimated from full trace export of the speckle tracking software and used together with the segmental wall thickness measurements to estimate segmental wall stress according to the formula of Laplace. Segmental stress-strain loops were generated, the area of which was considered to represent myocardial work per volume-unit [9]. Regional myocardial work in the septum and lateral wall was calculated as average of the stress-strain loop areas of the basal, mid, and apical segments of the respective wall.

### PET acquisition protocol

All patients underwent resting dynamic ^13^N-NH_3_ and static ^18^F-FDG PET studies (Biograph HiRez 16 PET/CT, Siemens, Erlangen, Germany) 1 week before CRT implantation (except for one patient who underwent a ^99m^Tc-tetrofosmin perfusion scintigraphy). A scout acquisition followed by a low-dose CT (80 kVp, 11 mAs) was performed for optimal patient positioning and subsequent CT-based attenuation correction of the PET emission data.

For ^13^N-NH_3_ PET, a 30-min dynamic list-mode acquisition was started together with a slow bolus intravenous administration of 10 MBq/kg ^13^N-NH_3_. In case of a 1-day protocol, the ^13^N-NH_3_ scan always preceded the ^18^F-FDG scan with a minimum interval of 60 min between tracer administrations.

^18^F-FDG PET scan was performed using the hyperinsulinemic euglycemic clamp technique in accordance with the method of Lewis et al. [11]. After reaching a steady-state plasma glucose level, 4.25 MBq/kg ^18^F-FDG was administered intravenously and a 40-min acquisition was performed approximately 45 min after tracer administration.

### Image processing

Before image reconstruction, alignment between PET and CT images was evaluated and manual realignment between both images was performed if deemed necessary by the investigator. All PET images were reconstructed using the ordered-subsets expectation maximization algorithms (4 iterations and 8 subsets), matrix size 256 × 256 and 5.0-mm Gaussian filter. Attenuation correction was performed using a low-dose CT scan.

^18^F-FDG PET static images were generated from the whole 40-min acquisition. ^13^N-NH_3_ list-mode file was rebinned into 22 frames (12 frames × 10 s, 4 frames × 30 s, 3 frames × 120 s, 1 frame × 180 s, 1 frame × 420 s, and 1 frame × 600 s).

### PET image analysis

All reconstructed ^13^N-NH_3_ and ^18^F-FDG PET images were analysed using in-house developed software [12]. Briefly, each myocardial image was resampled into 16 radial slices and a 17 segment polar map was generated according to previously validated methods [12, 13]. LV polar map was divided into 4 regions corresponding to the septal, lateral, anterior, and inferior wall with exclusion of the apex (segment 17). Analysis of perfusion and metabolism was performed focusing on the septal and lateral wall.

#### Absolute quantification of MBF with ^13^N-NH_3_

Absolute quantification of MBF per region was performed by modelling the first 10-min emission data of ^13^N-NH_3_ using a two-tissue compartment model [14]. Estimated rate constants were calculated using a weighted least-square method and were corrected for spillover and partial volume effect, as previously reported [14]. Because the amount of ^13^N-NH_3_ metabolites is known to increase after the first 2 min after tracer administration, metabolite correction was performed [15]. In addition to regional absolute MBF, septal-to-lateral wall MBF ratio (SLR) was calculated by dividing mean MBF in the septum by mean MBF in the lateral wall.

#### Analysis of ^18^F-FDG uptake

Regional ^18^F-FDG uptake was expressed as mean standardized uptake value (SUVmean). Septal to lateral wall ratio (SLR) was calculated by dividing mean ^18^F-FDG uptake in the septum by mean uptake in the lateral wall.

### Cardiac resynchronization therapy

CRT implantation was performed according to the guidelines [16]. In short, LV pacing leads were positioned, guided by coronary venography, preferably in the lateral and postero-lateral venous branches. After implantation, the device was set to bi-ventricular pacing in all patients.

### Statistical analysis

Statistical analysis was performed using SPSS Statistics 20 (IBM, Chicago, IL, USA). Shapiro-Wilk test was used to check the normality of data distribution. Normally distributed continuous variables were expressed as mean ± standard deviation; otherwise, median and interquartile range was used. Categorical variables were represented as percentages. Paired and unpaired *t* test with Bonferroni correction was used for comparison of continuous variables, while contingency tables were used for categorical variables. The correlation between parameters was assessed with Pearson correlation coefficients for normally distributed data; otherwise, Spearman coefficient was used. Interobserver and intraobserver variability of echocardiographic measurements of mechanical dyssynchrony (SF, AR) was performed in the whole study population using Kappa statistics. All statistical tests were two-tailed. A *p* value of less than 0.05 was considered statistically significant.

## Results

### Patient characteristics

Thirty patients (mean age 68 ± 10 years, 16 (53%) males) were included in the study. Coronary artery disease was excluded by late gadolinium enhancement CMR in 23/30 patients, by coronary angiography in 2/30 patients and all patients underwent thorough evaluation of patient history and complaints by an experienced treating cardiologist.

All patients had echocardiography pre-CRT. Two patients died during the first 6 months of follow-up so post-CRT echocardiography was available in only 28 patients. On pre-CRT echocardiography, 23 (77%) patients had mechanical dyssynchrony, while 7 (23%) patients had no mechanical dyssynchrony. Among patients with mechanical dyssynchrony, 2 patients had only AR, 1 patient had only SF, and 20 patients had both AR or SF. The QRS morphology on surface ECG in patients with mechanical dyssynchrony was represented mainly by LBBB (91%), while QRS pattern in patients without mechanical dyssynchrony was represented by LBBB (57%), non-specific intraventricular conduction delay (29%), and right ventricular pacing (1%). Clinical characteristics of our study cohort are presented in Table 1.

**Table 1:** Clinical characteristics of patients

Parameter	All patients (*n* = 30)	With mechanical dyssynchrony (*n* = 23)	Without mechanical dyssynchrony (*n* = 7)	*p* value
Clinical characteristics:				
Male (% of total)	16 (53%)	10 (43%)	6 (86%)	0.05
Age (years)	68 ± 10	68 ± 9	68 ± 12	0.94
Diabetes mellitus (% of total)	4 (13%)	3 (13%)	1 (14%)	0.93
Systolic blood pressure (mmHg)	134 ± 22	134 ± 21	120 ± 28	0.18
Diastolic blood pressure (mmHg)	69 ± 15	70 ± 14	65 ± 17	0.47
NYHA class II/III:	17 (57%)/13 (43%)	12 (52%)/11 (48%)	5 (71%)/2 (29%)	0.42
Electrocardiographic parameters:				
QRS width (ms)	161 ± 16	161 ± 15	163 ± 21	0.73
LBBB (Strauss)	25 (83%)	21 (91%)	4 (57%)	0.03
RV pacing	1 (3%)	0 (0%)	1 (14%)	0.23
NS-IVCD	4 (10%)	2 (9%)	2 (29%)	0.21
Echocardiographic parameters (pre-CRT):				
EDV (ml)	142 [133;193]	142 [126;203]	164 [116;202]	0.88
ESV (ml)	100 [91;138]	98 [85;147]	101 [76;138]	0.74
EF (%)	32 ± 8	32 ± 9	32 ± 5	0.99
Heart failure therapy:				
β-blockers	26 (87%)	19 (83%)	7 (100%)	0.54
ACEi/ARB	27 (90%)	21 (91%)	6 (86%)	0.66
Aldosterone antagonists	17 (57%)	13 (57%)	4 (57%)	0.9

*ACEi* angiotensin-converting enzyme inhibitors, *ARB* angiotensin-receptor blockers, *EDV* end-diastolic volume, *ESV* end-systolic volume, *LBBB* left bundle branch block, *RV* right ventricle, *NS-IVCD* non-specific intraventricular conduction delay

Three patients had suboptimal quality of ^18^F-FDG scan and 1 patient underwent ^99m^Tc-tetrofosmin perfusion scintigraphy instead of ^13^N-NH_3_ PET scan. After excluding these studies from the analysis, 27/30 and 29/30 patients have successfully completed respectively a ^18^F-FDG and ^13^N-NH_3_ PET scan. These scans were included in the further analysis independently of each other and were distributed between both patient groups as follows: 21 ^18^F-FDG and 22 ^13^N-NH_3_ scans belonged to the group of mechanical dyssynchrony while 6 ^18^F-FDG and 7 ^13^N-NH_3_ scans represented the group without mechanical dyssynchrony.

### Regional ^18^F-FDG uptake

**Fig. 1:**
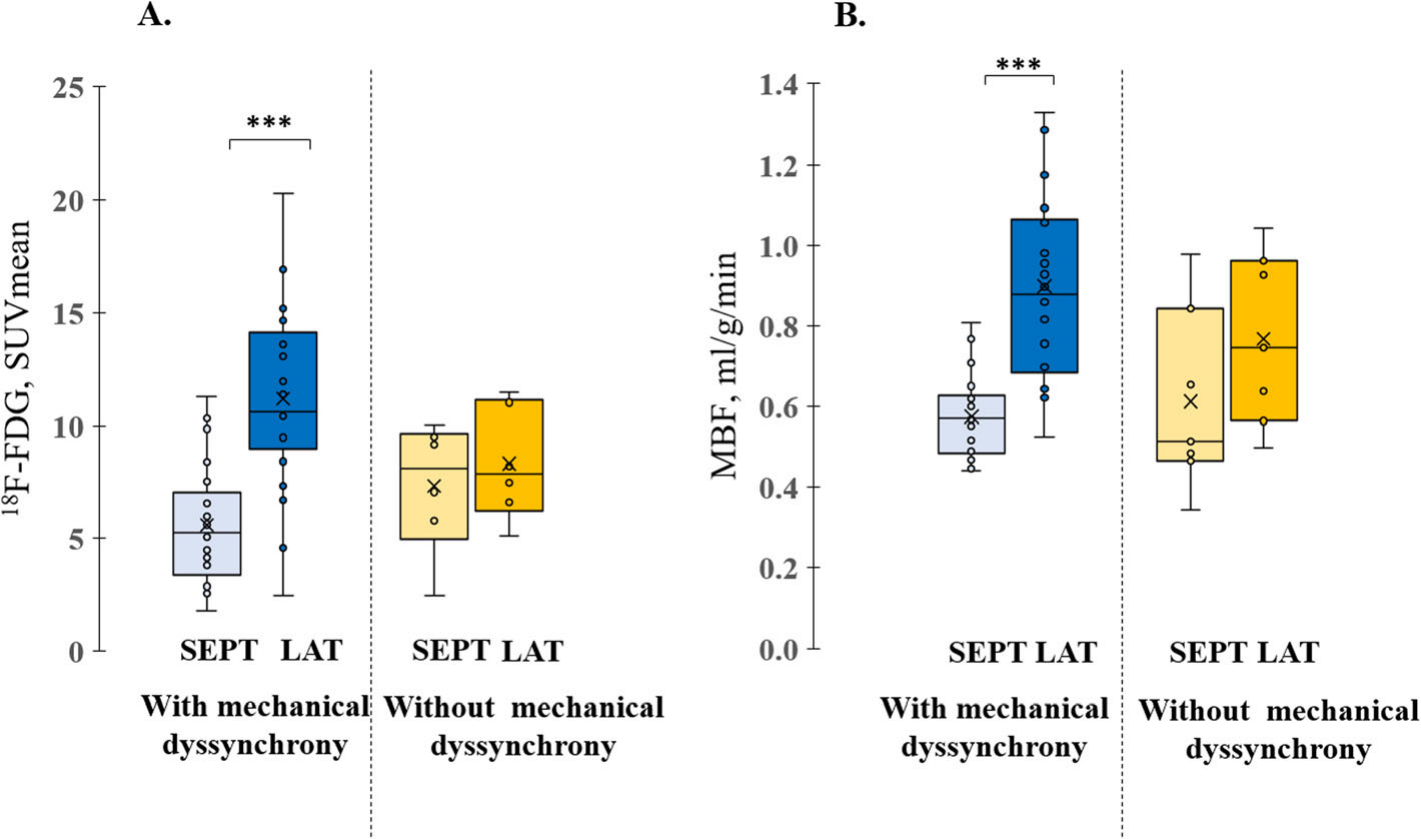
Regional glucose metabolism and myocardial blood flow (MBF). Regional glucose metabolism (^18^F-FDG uptake) (**a**) and MBF (**b**) in patients with and without mechanical dyssynchrony. SEPT, septum; LAT, lateral wall. ***p ≤ 0.0001

A significantly lower ^18^F-FDG SUVmean in the septum compared with the lateral wall was observed in the group with mechanical dyssynchrony (SUVmean 5.6 ± 2.7 vs 11.2 ± 4.1, respectively, *p* < 0.0001), while in the group without dyssynchrony, ^18^F-FDG distribution did not differ between septal and lateral wall (SUVmean 7.3 ± 2.9 vs 8.3 ± 2.5, respectively, *p* = 0.3) (Fig. 1a). ^18^F-FDG uptake in the lateral wall in patients with mechanical dyssynchrony was higher compared with those without, albeit not significantly (*p* = 0.1). Even though septal FDG uptake was lower in patients with mechanical dyssynchrony compared with patients without mechanical dyssynchrony, here there was no statistically significant difference between both groups (*p* = 0.22).

### Regional MBF

A significantly lower MBF in the septum compared with the lateral wall was observed in the group with mechanical dyssynchrony (0.6 ± 0.1 ml/g/min vs 0.9 ± 0.2 ml/g/min, respectively, *p* < 0.0001), while in the group without dyssynchrony, there was no statistical difference in the MBF between both walls (0.6 ± 0.2 ml/g/min vs 0.8 ± 0.2 ml/g/min, respectively, *p* = 0.16). No difference in regional MBF between both patient groups was observed (Fig. 1b).

### Relation between regional metabolism, MBF, and myocardial work

In the group with mechanical dyssynchrony, a significantly lower myocardial work was observed in the septum compared with the lateral wall (370 ± 816 mmHg*% vs 3174 ± 1033 mmHg*%, respectively, *p* < 0.0001), whereas no regional differences in myocardial work were observed in the group without mechanical dyssynchrony (2017 ± 685 mmHg*% vs 2267 ± 572 mmHg*%, respectively, *p* = 0.26). There was significantly less work performed by the septum in the group of dyssynchrony compared with the non-dyssynchrony group (370 ± 816 mmHg*% vs 2017 ± 685 mmHg*%, respectively, *p* = 0.0007). Additionally, negative (wasted) work was observed in the septum of 40% of patients with mechanical dyssynchrony, but never in patients without dyssynchrony. The work performed by the lateral wall was borderline significantly higher (*p* = 0.05) in the group with dyssynchrony compared with the group without dyssynchrony (3174 ± 1033 mmHg*% vs 2267 ± 572 mmHg*%, respectively).

**Fig. 2:**
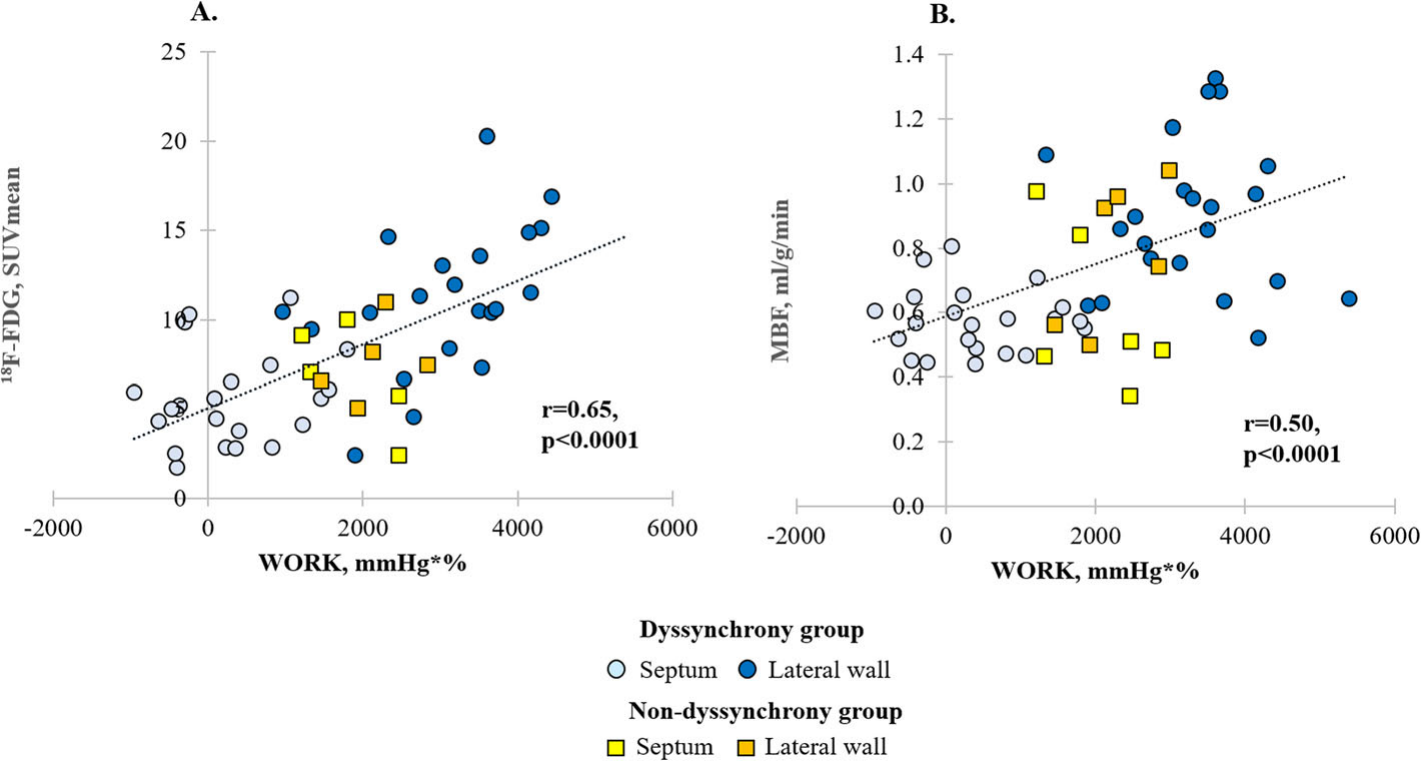
Correlation plots between myocardial work versus glucose metabolism and perfusion. Correlation between myocardial work and glucose metabolism (^18^F-FDG) (**a**) and myocardial work and MBF (**b**), measured in the septum and lateral wall in patients with and without mechanical dyssynchrony

**Fig. 3:**
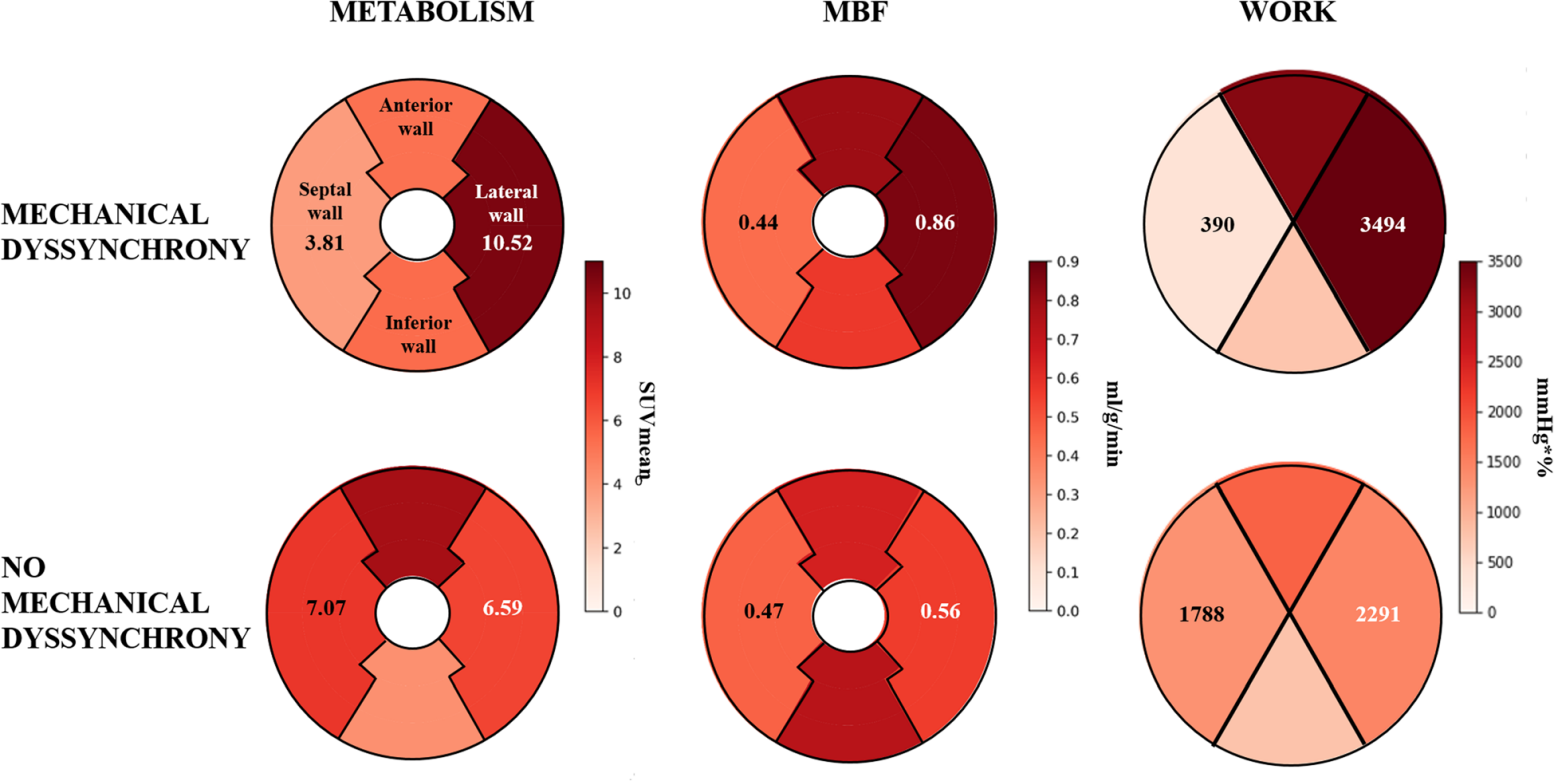
Representative example of the regional glucose metabolism, MBF, and workload. The figure shows lower septal compared with the lateral wall glucose metabolism, MBF, and workload in a patient with mechanical dyssynchrony and relatively homogeneous glucose metabolism, MBF, and workload in a patient without mechanical dyssynchrony

Both regional glucose metabolism and MBF linearly correlated with regional myocardial work (*r* = 0.65 and *r* = 0.5, respectively, *p* < 0.0001) (Fig. 2a, b). A representative example of the distribution of metabolism, perfusion, and work between septal and lateral wall in patients with and without mechanical dyssynchrony is shown in Fig. 3.

### Effect of mechanical dyssynchrony on ^18^F-FDG and MBF SLR and its relation to volumetric reverse remodeling

Patients with mechanical dyssynchrony demonstrated larger volumetric reverse remodeling 12 months after CRT compared with patients without mechanical dyssynchrony (Δ LV ESV 47 ± 14% vs − 3 ± 20%, *p* < 0.0001).

**Fig. 4:**
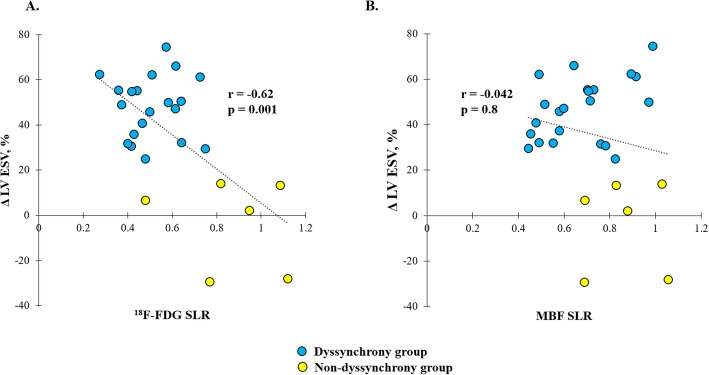
Correlation plots between volumetric reverse remodeling versus ^18^F-FDG and MBF SLRs. Correlation plot between ^18^F-FDG SLR versus Δ LV ESV (**a**) and MBF SLR versus Δ LV ESV (**b**) in patients with and without mechanical dyssynchrony

Patients with mechanical dyssynchrony compared with patients without mechanical dyssynchrony had a significantly lower ^18^F-FDG SLR (0.5 ± 0.1 vs 0.9 ± 0.2, respectively, *p* = 0.02). However, no differences between both groups were observed in MBF SLR (0.7 ± 0.2 vs 0.8 ± 0.2, respectively, *p* = 0.2). Pre-CRT ^18^F-FDG SLR showed a significant inverse correlation with volumetric reverse remodeling 12 months after CRT (*r* = − 0.62, *p* = 0.001) (Fig. 4a). Pre-CRT MBF SLR did not show a significant correlation with volumetric reverse remodeling (*r* = − 0.042, *p* = 0.8) (Fig. 4b).

### Reproducibility of assessment of mechanical dyssynchrony

Echocardiographic assessment of the presence of mechanical dyssynchrony demonstrated good intraobserver (*κ* = 0.85 (95% CI, 0.70–0.99), *p* < 0.0001) and interobserver (*κ* = 0.81 (95% CI, 0.64–0.98), *p* < 0.0001) agreement.

## Discussion

The main finding of our study in non-ischemic CRT candidates is the regional heterogeneity of myocardial glucose metabolism and perfusion in the presence of LV mechanical dyssynchrony, which can be attributed to the regional differences in myocardial loading conditions. Low ^18^F-FDG SLR appeared to be highly associated with the presence of mechanical dyssynchrony and showed a good inverse correlation with volumetric reverse remodeling after CRT.

### Regional myocardial ^18^F-FDG uptake

Our study showed that non-ischemic HF patients eligible for CRT, who present with mechanical dyssynchrony, have heterogeneous regional glucose metabolism with a twofold higher ^18^F-FDG uptake in the lateral wall compared with septum. In contrast, patients without mechanical dyssynchrony revealed an almost homogeneous metabolism.

Previous studies have demonstrated regional ^18^F-FDG uptake heterogeneity in the presence of ventricular conduction abnormalities. An experimental study of Ono et al. showed a significantly higher ^18^F-FDG uptake in the lateral wall compared with the septum (88.0 ± 5.2% versus 67.4 ± 12.1%) in right ventricular pacing [6]. Similar findings in patients with dilated cardiomyopathy and LBBB were reported by Nowak et al. [4]. However, regional glucose metabolism in the presence of mechanical dyssynchrony was not investigated in these studies; hence, a direct comparison with our findings cannot be performed.

Furthermore, Castro et al. showed marked overall LV ^18^F-FDG uptake heterogeneity in non-ischemic HF patients with mechanical dyssynchrony as evaluated by SPECT phase analysis [17]. This is in line with our findings, where patients with mechanical dyssynchrony showed pronounced differences in ^18^F-FDG uptake between septal and lateral wall, hence also overall LV heterogeneity. While in the study of Castro et al. all patients without mechanical dyssynchrony also demonstrated heterogeneity in LV metabolism, even though less pronounced, we could not confirm these findings in our study cohort. This discrepancy can be due to the small number of participants (*n* = 7) in our group of patients without dyssynchrony. However, our study population (23% without mechanical dyssynchrony) comes close to the real-world situation where about 30–35% CRT candidates do not present neither AR nor SF on echocardiography [3]. Additionally, the effect of using different modalities (SPECT vs. echocardiography) and approaches (automatic vs visual) for the assessment of mechanical dyssynchrony should not be neglected when comparing the results. Unfortunately, a more detailed comparison between both studies cannot be performed, as the study of Castro et al. did not explore LV metabolism per region.

### Regional absolute MBF

Similar to ^18^F-FDG findings, regional differences in MBF were pronounced only in the dyssynchrony patients while no differences were observed in the group without mechanical dyssynchrony.

There is a lot of controversy on regional myocardial perfusion in patients with ventricular conduction abnormalities, which may be attributed to the use of different imaging modalities, radiopharmaceuticals, study protocols, and approaches for flow assessment. The study by Baller et al. showed a low absolute MBF in the septal and high MBF in the lateral wall using ^11^C-acetate, while the study of Koepfli et al. showed a more homogeneous perfusion at rest using ^15^O-water [18, 19]. In the latter study, regional perfusion inhomogeneity was observed only during stress imaging which could be explained by the fact that ‘stress’ increases the imbalance in regional mechanical work resulting in higher regional perfusion changes [20]. Masci et al. reported a homogeneous ^13^N-NH_3_ MBF between LV walls in patients with LBBB and dilated cardiomyopathy, which may be explained by the inclusion of patients without advanced disease when LV dyssynchrony was not pronounced enough to induce perfusion heterogeneities as well as by the simplified approach of myocardial perfusion assessment [21].

However, also here, a direct comparison with our results cannot be performed, as abovementioned studies were not focusing on MBF in the presence of mechanical dyssynchrony, but rather analysed an overall population with ventricular conduction abnormalities irrespective of mechanical dyssynchrony. On the other hand, findings in the literature should be mainly driven by patients with mechanical dyssynchrony, as about 65% of patients with ventricular conduction abnormalities have at least one echocardiographic sign of mechanical dyssynchrony (AR or SF) [3]. Hence, decreased perfusion in the septum compared with lateral wall as described by aforementioned studies is in line with the heterogeneous regional perfusion in the dyssynchrony group reported in our study.

### Relation between regional metabolism, MBF, and myocardial work

Linear correlation between workload vs metabolism and workload vs MBF, demonstrated in our study, explains the different regional distribution patterns of both parameters between patients with and without mechanical dyssynchrony.

The patient group without mechanical dyssynchrony did not present any regional heterogeneity neither in myocardial work, nor in metabolism or MBF, suggesting that despite electrical dyssynchrony on ECG, all myocardial walls equally contribute to LV contraction, requiring comparable amount of energy and oxygen. In contrast, patients with mechanical dyssynchrony showed a pronounced regional heterogeneity in all three mentioned parameters with a clear shift of the septal values to the lower end and lateral wall towards the higher end of the spectrum. In this group of patients, septum does not significantly contribute to LV contraction, but rather wastes energy by stretching the opposite segments, whereas the lateral wall is the region which contributes the most to LV performance. The lateral wall, which is pre-stretched by the early-activated septum, contracts with a greater force (according to Frank-Starling law) and compensates for the zero external work of the septum. Such a redistribution of myocardial work, with unloading of the early-activated septum and a higher load in the late-activated lateral wall, causes a respective adaptation of regional energy and oxygen demand which explains the high metabolism and perfusion in the lateral wall and the low values in the septum observed in our study.

### Effect of mechanical dyssynchrony on SLRs and its relation to volumetric reverse remodeling

Despite the regional differences in both glucose metabolism and perfusion in patients with mechanical dyssynchrony, only ^18^F-FDG SLR and not MBF SLR was associated with the presence of mechanical dyssynchrony.

Several studies have demonstrated that the presence of mechanical dyssynchrony is favorable for volumetric CRT response [3, 22, 23]. However, the inconsistent results in the literature, controversy around the definition of mechanical dyssynchrony, and the lack of randomized trials have so far prevented the general acceptance and inclusion of mechanical dyssynchrony in CRT guidelines. On the other hand, relatively simple markers of mechanical dyssynchrony, such as AR and SF, used in our study and validated earlier, have been shown to be reliable and reproducible as surrogates for mechanical dyssynchrony [24]. Interestingly, in our study almost all patients with SF and/or AR had LBBB QRS morphology on surface ECG, while QRS patterns of non-dyssynchrony patients were more diverse and consisted of LBBB, right ventricular pacing, and non-specific intraventricular conduction delay. These findings demonstrate that LBBB is often, but not always, associated with mechanical dyssynchrony. This might be one of the reasons why not all patients fulfilling the current criteria for CRT implantation successfully respond to this therapy and highlights the need to define other parameters that may better identify patients who will most likely benefit from CRT.

In contrast, low ^18^F-FDG SLR is associated with the presence of at least one of these echocardiographic markers of mechanical dyssynchrony. Furthermore, ^18^F-FDG SLR showed a good inverse correlation with volumetric reverse remodeling 12 months after CRT implantation. Interestingly, the majority of patients with good reverse remodeling belonged to the group of mechanical dyssynchrony. Our findings highlight the existing relation between mechanical dyssynchrony, ^18^F-FDG SLR, and volumetric reverse remodeling and may indicate a place for nuclear imaging in prediction of CRT response. Further studies are needed to explore this assumption further, especially in ischemic patients or patients with a QRS duration between 120 and 150 ms, who still remain a major challenge to improve the response rate to CRT.

### Study limitations

Although ischemia was excluded in our patient cohort based on late gadolinium enhancement CMR and/or coronary angiography 3 months prior to CRT implantation and/or thorough clinical evaluation of patient history and complaints, one cannot completely rule out ischemia at the time of CRT implantation; however, these chances are negligible.

In the current study, clinically used reconstruction algorithms and software programs were applied without correcting for partial volume effect, which may lead to a slight underestimation of the true tracer concentration in the thinned LV walls.

We did not perform absolute quantification of cardiac glucose metabolism, but only glucose uptake defined by SUVmean was used in the current study. Quantification of glucose consumption as well as using other metabolic PET tracers such as ^11^C-acetate could help to better understand the pathophysiological mechanisms and metabolic adaptations during abnormal LV activation patterns.

Another limitation is the difference in LV segmentation models used for perfusion/metabolism (17 AHA model) and regional myocardial work (18 segment model). However, since regional values were represented as averages of corresponding segments and since the difference between both segmentation models is only the exclusion of the small apical region, we believe that the influence on the correlation between SLRs and work is negligible.

## Conclusions

Non-ischemic HF patients with mechanical dyssynchrony demonstrate heterogeneous regional glucose metabolism and MBF, while patients without mechanical dyssynchrony do not, which can be attributed to alterations in regional myocardial workload between both groups. ^18^F-FDG SLR appeared to be highly associated with the presence of mechanical dyssynchrony and should be explored as a possible predictor for favorable CRT response.

## Data Availability

The datasets used and/or analysed during the current study are available from the corresponding author on request

## Abbreviations

AR: Apical rocking
CRT: Cardiac resynchronization therapy
^18^F-FDG: ^18^F-fluorodeoxyglucose
HF: Heart failure
LBBB: Left bundle branch block
LV: Left ventricular
MBF: Myocardial blood flow
PET: Positron emission tomography
SF: Septal flash
SLR: Septal to lateral wall ratio
SUV: Standardized uptake values
